# Antiseptic Remedies in the Treatment of Diseases of the Eye and Ear

**Published:** 1883-09

**Authors:** E. L. Holmes


CHICAGO MEDICAL

JOURNAL & EXAMINER.

VOL. XLVII.—SEPTEMBER, 1883.—No. 3.

### Original Communications.

### ARTICLE I.

ANTISEPTIC REMEDIES IN THE TREATMENT OF DISEASES OF THE EYE AND EAR. From a Lecture at the Clinic of Rush Medical College. By DR. E. L. HOLMES.

A candid survey of the experience of a large number of specialists in the application of antiseptic remedies in ophthalmic and aural surgery, leads, I think, to the conclusion that, after all that is possible has been accomplished by pure water, the field is limited in which antiseptics are proven to be of decided benefit.

This field, though limited, is very important, and embraces the treatment of several serious conditions.

The habit of carefully washing the hands, and especially the finger nails, during the hours of practice, and of cleansing all instruments, brushes and sponges in an abundance of pure water immediately before and after use, nearly banishes the danger of septic poisoning.

Those instruments and dressings which have been neglected after operations, and which retain dried pus, blood and other discharges, should remain some time in hot water, and be thoroughly disinfected by means of a quite strong solution of carbolic acid.

Before every operation, however trivial, it is well to cleanse the integument of the lids and adjoining parts with a solution of boric acid or of carbolic acid.

It is safe to keep all sponges and dressings impregnated with a weak solution of one of these acids and closely confined in a tight vessel.

While I must urge upon you the neccssity of strict cleanliness in the management of surgical and of accidental wounds of the eye, I must admit, that within two or three years, I have accomplished this simply by the use of water. During more than twenty-four years I had never known a case of erysipelas, or of serious inflammation to follow an operation on the lids, or the operations for strabismus and pterygium, either in hospital or in private practice. And yet at the Eye and Ear Infirmary there had been several epidemics of facial erysipelas.

For the moral effect and for your own satisfaction in case of a possible accident, I advise you to apply, as dressing, compresses, moistened with antiseptic lotions, for all operations and wounds.

There is perhaps more certainty in regard to the benefits derived from the use of antiseptics in at least three forms of disease of the eye — purulent conjunctivitis, especially gonorrhœal— "creeping" ulcer of the cornea and blennorrhœa of the sack and nasal duct. In the first two diseases the eye may be frequently irrigated with the lotion; in the last, the lotion should be injected by means of a suitable syringe.

Some specialists believe that solutions of atropine, homatropine, duboisia, eserine and pilocarpine should be prepared as far as practicable only at the time they are required for use. A minute quantity of boric acid or of carbolic acid tends to prevent the growth of fungi in these solutions, which almost invariably appear without some form of germicide.

It will sometimes be within your power to prevent the spread of contagious diseases of the eye, by warning individuals suffering with purulent discharges from the eyes or genital organs, against the dangers to themselves and those around them resulting from want of strict cleanliness of person and clothing.

Special warning seems necessary in cases of vaginitis in young (scrofulous) children. Cases are not rare in which the disease has been overlooked or neglected, and the poison unwittingly conveyed by the children to their eyes.

As accoucheurs you may prevent serious disease of the eye in new-born infants by thoroughly irrigating with antiseptic solutions the vagina of every woman suffering from leucorrhœa during the last hour of parturition.

In families, schools, and public institutions, where many persons are collected, all patients with purulent discharges from the eyes should be carefully isolated. The lids of such patients must be kept clean by the frequent removal of pus by means of bits of linen or absorbent cotton. Pillow cases, towels and handkerchiefs soiled with pus should be placed at once in water, thus preventing contamination of the air from dried pus cells.

The most convenient solutions for the eye are perhaps the following: Carbolic acid, one grain to the ounce of water; boric acid, eight grains to the ounce; salicylic acid, three grains, with four grains of borax to the ounce; hyd. Bichlor., one grain to eight or ten ounces of water. It is doubtful if other agents posess special advantages over those above mentioned.

I need not dwell on the application of this class of remedies in diseases of the ear. Their special use is limited almost entirely to purulent affections. The strictest cleanliness of the external meatus, as attained by the frequent and prolonged injections of warm water, must be enjoined in obstinate cases; the meatus, then carefully dried by means of cotton, is to be partially filled with powdered boric acid, or the usual astringents. It is seldom that patients will not easily tolerate injections of warm water. Exceptionally the cotton and cotton holder mus. be employed to remove pus from the ear.

Following the injection, in place of the boric acid may be used, in certain cases, a solution of carbolic acid, of permanganate of potash, or of thymol, alternated with astringent solutions.

The utmost care is demanded in cleansing instruments employed for the ear, as for the eye, immediately after use. Pure water, we believe, is usually sufficient for this purpose. Eustachian catheters and other instruments introduced into the nostrils and throat are liable to come in contact with poisonous discharges. For this reason they should be placed for a time in quite a strong solution of carbolic acid. Except when instruments have been neglected after use, it seems to be excess of caution to boil them in hot water.

I do not wish to consider at this time the manner in which antiseptic remedies are beneficial, nor to discuss the theories whether organisms are the cause or the result of diseases of the eye and ear.

